# Conserved requirement for DEAD-box RNA helicase Gemin3 in *Drosophila *oogenesis

**DOI:** 10.1186/1756-0500-5-120

**Published:** 2012-02-23

**Authors:** Ruben J Cauchi

**Affiliations:** 1 Department of Physiology and Biochemistry, Faculty of Medicine & Surgery, University of Malta, Msida MSD 2080, Malta G.C

**Keywords:** *Drosophila*, Gemin3, Germline, Oogenesis, Spinal muscular atrophy, Survival of motor neurons

## Abstract

**Background:**

DEAD-box RNA helicase Gemin3 is an essential protein since its deficiency is lethal in both vertebrates and invertebrates. In addition to playing a role in transcriptional regulation and RNA silencing, as a core member of the SMN (survival of motor neurons) complex, Gemin3 is required for the biogenesis of spliceosomal snRNPs (small nuclear ribonucleoproteins), and axonal mRNA metabolism. Studies in the mouse and C. *elegans *revealed that loss of Gemin3 function has a negative impact on ovarian physiology and development.

**Findings:**

This work reports on the generation and characterisation of *gemin3 *mutant germline clones in *Drosophila *adult females. Gemin3 was found to be required for the completion of oogenesis and its loss led to egg polarity defects, oocyte mislocalisation, and abnormal chromosome morphology. Canonical Cajal bodies were absent in the majority of *gemin3 *mutant egg chambers and histone locus bodies displayed an atypical morphology. snRNP distribution was perturbed so that on *gemin3 *loss, snRNP cytoplasmic aggregates (U bodies) were only visible in wild type.

**Conclusions:**

These findings establish a conserved requirement for Gemin3 in *Drosophila *oogenesis. Furthermore, in view of the similarity to the phenotypes described previously in *smn *mutant germ cells, the present results confirm the close functional relationship between SMN and Gemin3 on a cellular level.

## Background

The DEAD-box RNA helicase Gemin3 (also referred to as DP130 or DDX20) is a core member of the SMN (survival of motor neurons) complex [1]. The latter has been linked to the neuromuscular degenerative disease SMA (spinal muscular atrophy) through decreased levels of the eponymous SMN protein [2]. Gemin3 exhibits considerable sequence similarity to other DEAD-box family members within the nine motifs that form its 'helicase' core. Its flanking C-terminal domain is however highly divergent and is thus thought to provide specificity of function through specific substrate interactions. DEAD-box RNA helicases or unwindases are capable of rearranging inter- or intra-molecular RNA (ribonucleic acid) structures as well as dissociating or associating RNA-protein complexes [3]. *In vitro *studies revealed that in the case of Gemin3, the RNA helicase activity has a 5' to 3' directionality and is ATP-dependent [4]. In view of its job description, Gemin3 fits in with the most-documented role of the SMN complex, namely the assembly of Sm protein cores onto snRNAs (small nuclear RNAs) in the cytoplasm to generate snRNPs [small nuclear RNPs (ribonucleoproteins)] that are then shuttled to the nucleus where they participate in pre-mRNA splicing following maturation in the Cajal body [1,5]. Gemin3 is also an active participant as a component of the SMN complex in an emerging neuronal-specific role that is independent of snRNP assembly [6,7]. In this respect, SMN and select Gemins including Gemin3 were found to significantly co-localise and associate with β-actin mRNA in neurite granules hinting at a function for the SMN complex in the assembly and/or transport of localised mRNP complexes [8-10].

Gemin3 is a leading example of the multifunctional nature of RNA helicases [1,3]. It was originally isolated as a cellular factor that associates with Epstein-Barr virus nuclear proteins EBNA2 and EBNA3C, which play a role in the transcriptional regulation of latent viral and cellular genes [11]. Extending the role of Gemin3 in transcription, later studies reported on its ability to interact with and modulate the activity of various transcription factors including SF-1 (steroidogenic factor 1) [4,12], Egr2 (early growth response protein 2) [13], FOXL2 (forkhead transcription factor) [14], and METS (mitogenic Ets transcriptional suppressor) [15]. Furthermore, Gemin3 forms a less abundant complex with Gemin4, AGO2 (Argonaute2) and numerous miRNAs (microRNAs), which importantly is independent of the SMN complex [16]. miRNAs function within the RISC (RNA-induced silencing complex) to repress translation of intracellular mRNAs having complementary nucleotide sequences and in this regard, a multiprotein RISC including as its components AGO2, FMRP (fragile X mental retardation protein), p100 and Gemin3, was shown to form *in vivo *in response to treatment with specific short interfering RNAs [17]. Although the ATPase/RNA helicase activity of Gemin3 does not appear to be required for its function as a transcriptional regulator, Gemin3 probably acts as a bone fide RNA helicase in RNP metabolism and RNA silencing.

Disrupting a gene of interest allows us to garner valuable information on the gene's cellular functions. *Gemin3 *is an essential gene since its loss leads to lethality in both vertebrates and invertebrates [18-20]. A role in snRNP assembly was confirmed on intracellular reduction of Gemin3 in HeLa cells either via proteolysis by the poliovirus-encoded proteinase 2A^pro ^or siRNA (small interfering RNA)-mediated knockdown, both of which were found to independently disrupt Sm core assembly [21,22]. Although homozygous mouse knockout of *Gemin3 *leads to early embryonic lethality, heterozygous Gemin3 knockout female mice have defects in ovarian morphology and function [18]. Interestingly, a germline function was corroborated by Minasaki et al. [19] who isolated a mutant allele of the *C. elegans Gemin3 *gene orthologue *mel-46 (maternal effect lethal-46)*, which causes variable defects in oogenesis in viable homozygous mutant hermaphrodites. The progeny of the latter is however embryonic lethal and *mel-46 *homozygous knockout results in larval lethality. In *Drosophila*, maternal contribution prolongs survival of *gemin3 *loss-of-function mutants to the late larval stages, where they develop mobility and neuromuscular junction defects before death [20,23]. Aiming at establishing whether the role of Gemin3 in oogenesis is conserved in *Drosophila*, this work reports on the generation and characterisation of *gemin3 *mutant germline clones.

## Materials and methods

### Fly genetics

*Drosophila melanogaster *stocks were cultured on standard molasses/maize meal and agar medium in plastic vials at 25°C. The wild type fly strain was the *y w *stock. The mutant strain was *gemin3^R ^*(P[PZ]Dhh1^rL562^), which is a recessively lethal transposon insertion allele and was described in a previous study [20].

### Generation of *gemin3 *mutant germline clones

Generation of germline clones relied on the use of the FLP-DFS (yeast flippase-dominant female sterile) technique [reviewed in [24]]. An FRT (flippase recombinase target) site was recombined onto the *gemin3^R ^*mutant chromosome using established genetic cross schemes. To generate *gemin3^R ^*germline clones, virgin females having the *w; gemin3^R ^FRT2A/TM6B Tb^1 ^*genotype were crossed to *y w hsFLP; ovo^D1 ^FRT2A/TM3 Ser *males and recombination in the resulting progeny was stimulated through heat-shock at 37°C for 1 h at day 3, 4, and 5 after egg hatching. Egg chambers that survive beyond stage 4 in the ovaries of the female offspring (*y w hsFLP; gemin3^R ^FRT2A/ovo^D1 ^FRT2A*) lack *ovo^D1 ^*and are hence homozygous for *gemin3^R^*. Reintroduction of the full-length *gemin3 *transgene in mutant germline clones was attempted to assess phenotype rescue but was unsuccessful.

### Immunofluorescence staining

Ovaries of female flies, which were fed on wet yeast for ~3 days in the presence of males, were dissected in PBS (phosphate buffered saline) at room temperature and later fixed in 4% paraformaldehyde in PBS. The tissue samples were then washed for 30 min in 1× PBS + 0.3% Triton^® ^X-100 + 0.5% normal goat serum following removal of the fixative, and subjected to overnight incubation with primary antibodies at room temperature. The following day, the tissues were stained with anti-mouse or anti-rabbit Alexa Fluor^®^-conjugated secondary goat antibodies. Tissues were finally counterstained with Hoechst 33342 nuclear stain prior to washing and mounting in 80% glycerol in PBS. Zeiss LSM 510 META or Bio-Rad Radiance 2100 confocal microscopes were used for imaging tissues. Primary antibodies used include rabbit anti-coilin (gift from Joseph Gall, Carnegie Institution, Baltimore, MD, U.S.A. [25]), rabbit anti-Lsm11 (gift from Joseph Gall, Carnegie Institution, Baltimore, MD, U.S.A. [26]), rabbit anti-eIF4E (eukaryotic translation initiation factor 4E) (gift from James Wilhelm, University of San Diego, San Diego, CA, U.S.A. [27]), rabbit anti-DCP1 (decapping protein 1) (gift from James Wilhelm, University of San Diego, San Diego, CA, U.S.A. [28]), and mouse anti-TMG (2,2,7-trimethylguanosine) (Calbiochem, Merck KGaA, Darmstadt, Germany). The original confocal images were processed using the ImageJ software (National Institutes of Health, Bethesda, MD, U.S.A.).

### Findings

**Figure 1:**
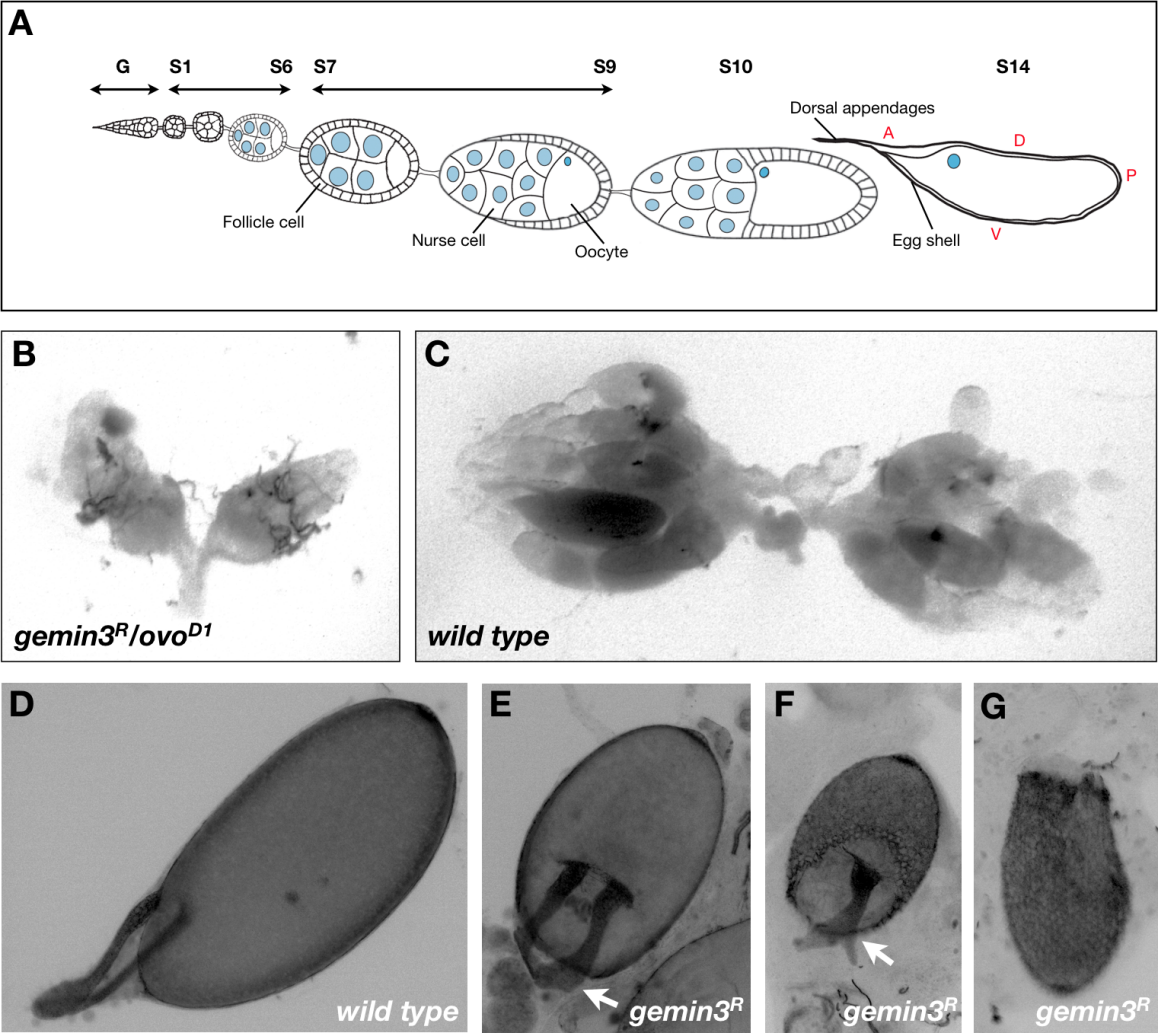
**Ovarian and egg polarity defects in the absence of Gemin3**. (A) Schematic of the sequential stages (S1 to S14) of egg chamber development after budding off the germarium (G) [adapted from [30]]. A mature egg (S14) exhibits asymmetry along its A-P and D-V axes. (B, C) Ovaries of *gemin3^R^/ovo^D1 ^*female flies are shrivelled in contrast to wild type, a phenotype resulting from the early developmental arrest of *gemin3^R ^*homozygous germ cells. Images are of the same magnification. (D-G) Compared to wild type, *gemin3 *mutant eggs are smaller in size and display a range of DA phenotypes including DAs, which are broader and shorter compared to wild type (E, arrow) as well as forked DAs that are fused at their base (F, arrow). Sometimes eggs display a net-like chorion or eggshell with no apparent D-V polarity. In D to G, images are of the same magnification.

The null allele *gemin3^R ^*was the mutant strain selected for the generation of homozygous *gemin3 *mutant germline clones based on earlier data demonstrating that it is the only allele available whose recessive lethality is specific to *gemin3 *disruption [20]. After recombining a flippase recombinase target (FRT) site onto the *gemin3^R ^*mutant chromosome, female flies with a *gemin3^R ^FRT/ovo^D1 ^FRT *genotype were subjected to flippase-directed recombination during development so that their ovaries become populated by recombinant homozygous *gemin3^R^*, recombinant homozygous *ovo^D1 ^*and non-recombinant heterozygous *gemin3^R^*/*ovo^D1 ^*germ cells. *Drosophila *ovaries are organised into 16-20 hollow tubular structures, called ovarioles, which contain progressively maturing egg chambers. Each egg chamber is composed from a single oocyte, 15 nurse cells that supply the developing oocyte with synthesised mRNAs as well as proteins, and a surrounding monolayer of follicle cells (Figure 1A). After budding off the germarium, further development of the egg chamber is usually divided into 14 stages based on morphological criteria, where stage 1 refers to a newly formed egg chamber and stage 14 is a mature egg that is ready to be fertilised and oviposited [29]. Ovaries of *gemin3^R ^FRT/ovo^D1 ^FRT *female flies were smaller compared to wild type (Figure 1B-C), a phenotype that cannot be explained solely by the presence of the dominant *ovo^D1 ^*mutation, which limits germ cell development up to stage 4. Indeed, the majority of post-stage 4 egg chambers, which are homozygous for the *gemin3^R ^*mutant allele, arrested their development around stage 6-7 prior to degeneration and hence are mostly responsible for the shrivelled *gemin3^R^/ovo^D1 ^*ovaries. These observations clearly show that Gemin3 is required for completion of oogenesis.

Although very infrequently observed, stage 14 eggs derived from *gemin3^R ^*germline clones had a smaller size compared to wild type, were sterile since no hatched progeny was apparent upon crossing the females to wild type *Oregon R *males, and displayed polarity defects (Figure 1D-G). The wild type mature *Drosophila *egg is visibly asymmetric along its A-P (anterior-posterior) and D-V (dorsal-ventral) axes. It is in fact characterised by two DAs (dorsal respiratory appendages) at the anterior end and a surface, which is more curved on the ventral than on the dorsal side (Figure 1D). DAs of *gemin3 *mutant eggs were shorter and more flattened compared to wild type, a phenotype observed in weakly dorsalised and lateralised eggs (Figure 1E). Furthermore, weakly ventralised eggs with two forked DAs fused at their base were sometimes observed (Figure 1F). Eggs that exhibit a net-like chorion or eggshell with no apparent D-V polarity were also occasionally obvious (Figure 1G).

Germline stem cells divide asymmetrically to produce a new stem cell and a cystoblast. The cystoblast undergoes four incomplete divisions to generate a cyst of 16 cells, one of which becomes the oocyte whilst the remaining differentiate into nurse cells [29]. Anchoring of the oocyte to the posterior of the egg chamber occurs during early oogenesis and relies on Cadherin-dependent adhesion between the oocyte and the posterior follicle cells [31,32].

**Figure 2:**
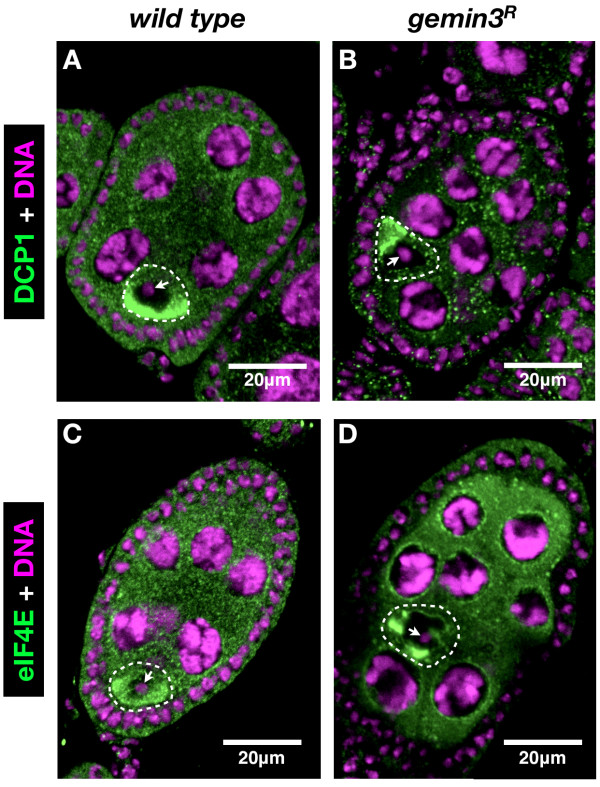
**Oocytes are incorrectly positioned on loss of *gemin3***. Compared to wild type in which the oocyte is always found at the posterior of stage 7 egg chambers, in the absence of *gemin3*, the oocyte was frequently misplaced. Egg chamber posterior is bottom left. DCP1 (A, B) and eIF4E (C, D) were used to identify the oocyte since they are expressed in all germline cells but accumulate in the oocyte (surrounded by a broken line with arrow pointing to the karyosome).

Upon staining for proteins enriched in the oocyte, the majority of egg chambers derived from *gemin3^R ^*germline clones were found to have mislocalised oocytes. Indeed, although *gemin3 *mutant oocytes were correctly specified as demonstrated by the condensation of their DNA into a small compact structure known as the karyosome, and the cytoplasmic enrichment of eIF4E as well as DCP1, the oocyte was found at a position other than the wild type posterior (Figure 2).

**Figure 3:**
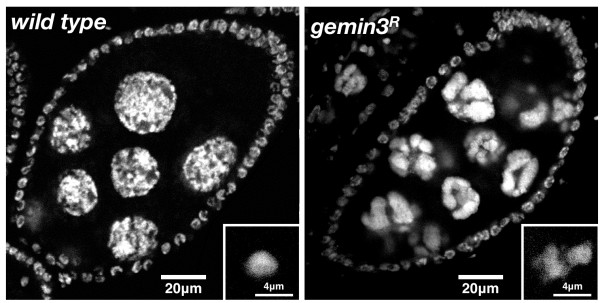
**Defective chromatin organisation in post-stage 5 *gemin3 *mutant egg chambers**. Hoescht-stained stage 8 egg chambers (posterior is top right) showing that the nurse cell chromosomes of *gemin3^R ^*egg chambers retain a compact 'blob-like' morphology, instead of dissociating and decondensing to be uniformly distributed throughout the nucleus as is typical in wild type. Insert shows Hoescht-stained oocyte nucleus, which is frequently decondensed in the mutant in contrast to the wild type where it is typically condensed into a small round karyosome.

During oogenesis, nurse cells undergo 10-12 endocycles, which are modified cell cycles that alternate between the S (DNA synthesis) and G (gap) phase. During the first four endocycles, homologous chromosomes are paired and progressively condense so that by stage 4, they acquire the banding pattern characteristic of polytene chromosomes. During the fifth endocycle, homolog pairing loosens but chromatin compaction increases so that by stage 5, banding is lost but five distinct chromatin masses corresponding to individual chromosome arms transiently appear as 'blobs' inside nurse cell nuclei. By stage 6, the chromosomes dissociate into 32 pairs of chromatids so that for the rest of oogenesis, the chromosomes are diffuse and uniformly distributed throughout the nucleus, with such a change in chromosome organisation postulated to facilitate the high transcription levels of components required for oocyte growth. During subsequent endocycles, the chromatids undergo incomplete DNA replication to generate 'subpolytene' chromosomes [33]. In *gemin3^R ^*germline clone-derived nurse cells, the five-blob chromosome morphology persisted in all mutant nurse cell nuclei well beyond stage 6 so that the chromosomes never assumed a uniformly dispersed distribution that is typical in wild type (Figure 3). Mutant oocyte nuclei were also found to have an abnormal morphology. Once their fate has been specified, wild type oocytes arrest in meiotic prophase and condense their DNA into a small compact structure known as the karyosome. Compared to wild type, decondensed karyosomes were observed in about half of the oocytes in *gemin3^R ^*homozygous germline clones.

**Figure 4:**
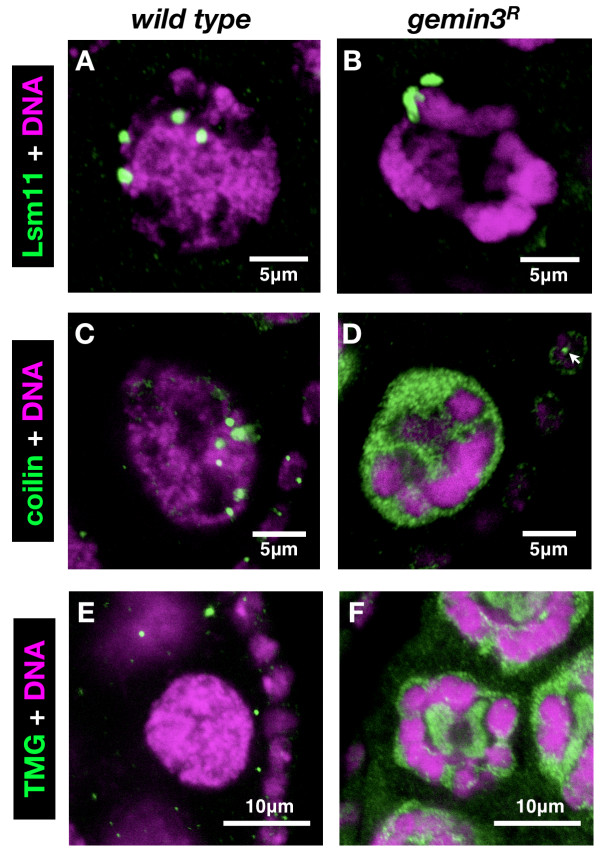
**Histone locus, Cajal and U bodies in *gemin3 *mutant germline clones**. (A, B) On staining for histone locus body marker Lsm11 (a component of the U7 snRNP), histone locus bodies were dispersed in multiple discrete loci throughout the wild type nurse cell nuclei. In contrast, in *gemin3^R ^*mutant nuclei, histone locus bodies appear as single aggregates attached to the condensed chromatin. Note that due to the 3D nature of the nucleus and the single sections demonstrated here, the *gemin3^R ^*mutant histone locus body aggregates sometimes appear as multiple foci, which in reality are a single structure. (C, D) Wild type polyploid nurse cell nuclei display several small coilin-rich Cajal bodies. In the majority of *gemin3^R ^*mutant egg chambers, this staining pattern is absent though coilin adopts a diffuse nuclear stain. Note that although coilin foci were absent in mutant nurse cell nuclei, they can still be detected in wild type follicle cells (arrow) that envelop the mutant egg chamber, thus highlighting successful immunostaining. (E, F) TMG-stained snRNPs are concentrated in discrete spherical structures (U bodies) within the cytoplasm of wild type egg chambers. In contrast, snRNP distribution is diffuse and exhibits nuclear predominance in *gemin3^R ^*germline clones.

Histone locus bodies, which contain factors required for processing histone pre-mRNAs including the U7 snRNP, are invariably associated with the histone gene cluster located on chromosome 2 and, as expected, increase in number once nurse cell nuclei polyploidise [25,34,35]. Stained for U7 snRNP component Lsm11, histone locus bodies were uniformly distributed in discrete circular structures throughout the nucleus in wild type nurse cells (Figure 4A). In contrast, *gemin3 *mutant nurse cell nuclei mostly exhibited a single histone locus body aggregate attached to the condensed chromatin, most probably chromosome arm 2L where the histone gene cluster resides (Figure 4B), a finding that further confirms the failed chromosome dispersal phenotype in mutant nurse cell nuclei.

Cajal bodies are sites for the assembly and/or posttranscriptional modification of several RNP species including snRNPs [5,34]. Coilin is one of the signature markers of Cajal bodies, which also host SMN complexes although under certain conditions these separate from Cajal bodies to form gems [36,37]. In wild type polyploid nurse cell nuclei, coilin-stained Cajal bodies form several small bright foci throughout the nurse cell nucleus (Figure 4C). This localisation pattern, which was also described previously [25,35], was absent in about 65% (n = 18) of *gemin3 *mutant germline-derived egg chambers, where coilin was diffusely distributed throughout the nurse cell nucleus instead of being enriched in discrete nuclear foci as is the case in wild type (Figure 4D).

In the cytoplasm, SMN complexes and uridine-rich snRNPs often aggregate in U bodies, which are thought to play a role in snRNP assembly and/or storage [1,26,38-40]. On staining for their TMG cap, snRNPs are concentrated in discrete spherical structures dotting the wild type nurse cell cytoplasm (Figure 4E). This typical U body pattern was not observed in *gemin3^R ^*mutant egg chambers, where snRNPs were diffusely distributed and had a nuclear predominance (Figure 4F).

In sum, the findings reported here extend a role for Gemin3 in ovarian physiology and development - reported earlier in mouse [18] and worm [19] - to *Drosophila*, hence suggesting that this function has been conserved throughout evolution. Interestingly, some of the germline *gemin3 *mutant phenotypes reported here were similar to those described previously in *smn *mutant germ cells in *Drosophila *[41], hence strengthening the view that SMN and Gemin3 function in a common cellular pathway and loss of either can lead to its disruption. It is tempting to speculate that this pathway involves assembly of snRNPs, the quantity and quality of which influences pre-mRNA splicing. Supporting this view, several SMN complex members including SMN and Gemin3 were shown to be required for snRNP biogenesis *in vitro *[21,22,42]. Furthermore, pre-mRNA splicing defects in transcripts of several functionally diverse genes were reported in SMN deficient mouse tissues [43,44] and snRNA levels were recently shown to be reduced in *smn *mutant *Drosophila *brain clones [45]. The lack of canonical Cajal and U bodies in *gemin3 *mutant germline clones is the strongest indicator of a failure in snRNP biogenesis. In this context, U bodies were not visible in the absence of *smn *[41], canonical Cajal bodies were lost in HeLa cells depleted of several components within the snRNP biogenesis pathway [46], and the number of Cajal bodies in motor neurons of a severe SMA patient was drastically decreased [47], hence suggesting that ongoing snRNP assembly is required for the integrity of both Cajal and U bodies. Interestingly, ovarian phenotypes similar to those uncovered in *gemin3 *and *smn *mutant germline clones [this study; [41]] were also observed in mutants of splicing factors Half pint and Hrb27C [48,49], thereby reinforcing the crucial role of pre-mRNA splicing in ovarian physiology and development.

## Conclusions

In addition to a motor function [20,23], the findings uncovered in the adult mutant female germline demonstrate that Gemin3 has a conserved function in oogenesis. Furthermore, the similar phenotypes observed in germ cells derived from the respective mutant clones point to a close functional relationship - possibly involving snRNP biogenesis - between SMN and Gemin3 on a cellular level at least within the germline. Given that a reduction in SMN levels is linked with motor defects in the form of SMA, it would be interesting to explore this pathway in tissues linked to motor function.

## Abbreviations

A-P: anterior-posterior; D-V: dorsal-ventral; DA: dorsal respiratory appendage; DCP1: decapping protein 1; eIF4E: eukaryotic translation initiation factor 4E; FRT: flippase recombinase target; mel-46: maternal effect lethal-46; RNA: ribonucleic acid; RNP: ribonucleoprotein; SMA: spinal muscular atrophy; SMN: survival of motor neurons; snRNA: small nuclear RNA; snRNP: small nuclear RNP; TMG: 2,2,7-trimethylguanosine.

## Competing interests

The author declares that he has no competing interests.

## Authors' contributions

RJC conceived, designed and performed the experiments. RJC analysed the data and wrote the manuscript. The author read and approved the final manuscript.
